# Extended and replicated white matter changes in obesity: Voxel-based and region of interest meta-analyses of diffusion tensor imaging studies

**DOI:** 10.3389/fnut.2023.1108360

**Published:** 2023-03-07

**Authors:** Lorielle M. F. Dietze, Sean R. McWhinney, Joaquim Radua, Tomas Hajek

**Affiliations:** ^1^Department of Psychiatry, Dalhousie University, Halifax, NS, Canada; ^2^Department of Medical Neuroscience, Dalhousie University, Halifax, NS, Canada; ^3^Institut d'Investigacions Biomèdiques August Pi i Sunyer (IDIBAPS), University of Barcelona, Barcelona, Spain; ^4^Mental Health Research Networking Center (CIBERSAM), Madrid, Spain; ^5^Department of Psychosis Studies, Institute of Psychiatry, Psychology, and Neuroscience, King's College London, London, United Kingdom; ^6^Department of Clinical Neuroscience, Center for Psychiatric Research and Education, Karolinska Institutet, Stockholm, Sweden; ^7^National Institute of Mental Health, Prague, Czechia

**Keywords:** obesity, white matter microstructure, fractional anisotropy, diffusion tensor imaging, magnetic resonance imaging, meta-analysis

## Abstract

**Introduction:**

Obesity has become a global public health issue, which impacts general health and the brain. Associations between obesity and white matter microstructure measured using diffusion tensor imaging have been under reviewed, despite a relatively large number of individual studies. Our objective was to determine the association between obesity and white matter microstructure in a large general population sample.

**Methods:**

We analyzed location of brain white matter changes in obesity using the Anisotropic Effect Size Seed-based d Mapping (AES-SDM) method in a voxel-based meta-analysis, with validation in a region of interest (ROI) effect size meta-analysis. Our sample included 21 742 individuals from 51 studies.

**Results:**

The voxel-based spatial meta-analysis demonstrated reduced fractional anisotropy (FA) with obesity in the genu and splenium of the corpus callosum, middle cerebellar peduncles, anterior thalamic radiation, cortico-spinal projections, and cerebellum. The ROI effect size meta-analysis replicated associations between obesity and lower FA in the genu and splenium of the corpus callosum, middle cerebellar peduncles. Effect size of obesity related brain changes was small to medium.

**Discussion:**

Our findings demonstrate obesity related brain white matter changes are localized rather than diffuse. Better understanding the brain correlates of obesity could help identify risk factors, and targets for prevention or treatment of brain changes.

## 1. Introduction

Obesity has become a major public health issue due to its extremely high prevalence. For instance, 63.1% of the population in Canada and 52% of adult population worldwide can be classified as overweight or obese (1, 2). Obesity is an established health risk factor for hypertension, type 2 diabetes, cardiovascular disorders, metabolic syndrome, and cancer among others (3–8) and is associated with massive health care costs and human suffering. It is less well recognized that the brain is one of the targets of obesity related damage (9, 10), and consequently, obesity is also associated with a range of brain disorders, including psychiatric (11), and neurodegenerative disorders/dementia (12–14).

It is important to map and better understand the brain correlates of obesity. Such studies could help identify risk factors for brain alterations, targets, and possibly new mechanisms of action for prevention or treatment of brain changes and associated cognitive or mental health outcomes. For instance, deterring obesity could prevent about 3% of depressive disorders, deterring maternal pre-pregnancy obesity would prevent about 9% of ADHD, and deterring maternal overweight pre/during pregnancy would prevent about 6% of both ADHD and autism spectrum disorders (15). Preliminary evidence suggests that weight loss is associated with improvements in brain structure (16).

Obesity is associated with diffuse changes in gray matter, including the medial prefrontal cortex, temporal pole, precentral gyrus, inferior parietal cortex and the cerebellum (17). The associations between obesity and brain gray matter are supported by several large studies (18–20). Some studies have documented an association between obesity and white matter integrity, but these findings are much less diffuse, less replicated, and under reviewed. A previous narrative review mentions links with obesity and alterations of white matter integrity in the genu, body, and splenium of the corpus callosum, fornix, cingulum, corona radiata, corticospinal tracts, uncinate fasciculus, and cerebellar peduncles (21). However, considering the heterogeneity of these findings, a meta-analysis is needed to quantitatively review the available evidence. Whole brain DTI studies can be analyzed using spatial meta-analysis, which identifies the most replicated location of obesity related alterations (22, 23). Region of interest studies can be used in a traditional effect size meta-analysis to establish the regional extent of obesity related alterations (24). Furthermore, focusing on replications among the two meta-analyses minimizes the risk of false positives (25).

A single previous spatial meta-analysis investigated the location of obesity related white matter alterations. However, this study had restrictive inclusion criteria and contained only one half of the available studies in the literature (26). Furthermore, regions of interest studies were not included in this meta-analysis, thus representing less than a third of the reviewed literature. There are no prior effect size meta-analyses quantifying the extent of obesity related alterations and no studies combining a spatial and effect size meta-analysis.

Thus, we present the first study that analyzes the location of white matter changes in obesity using a voxel-based spatial meta-analysis and then validates the results and estimates the extent of obesity-related alterations with a region of interest (ROI) effect size meta-analysis. This approach allowed us to review the entire available literature on this topic, maximize sample size, minimize false negatives and by focusing on replications to also minimize the risk of false positives.

## 2. Materials and methods

### 2.1. Search strategy

**Figure 1:**
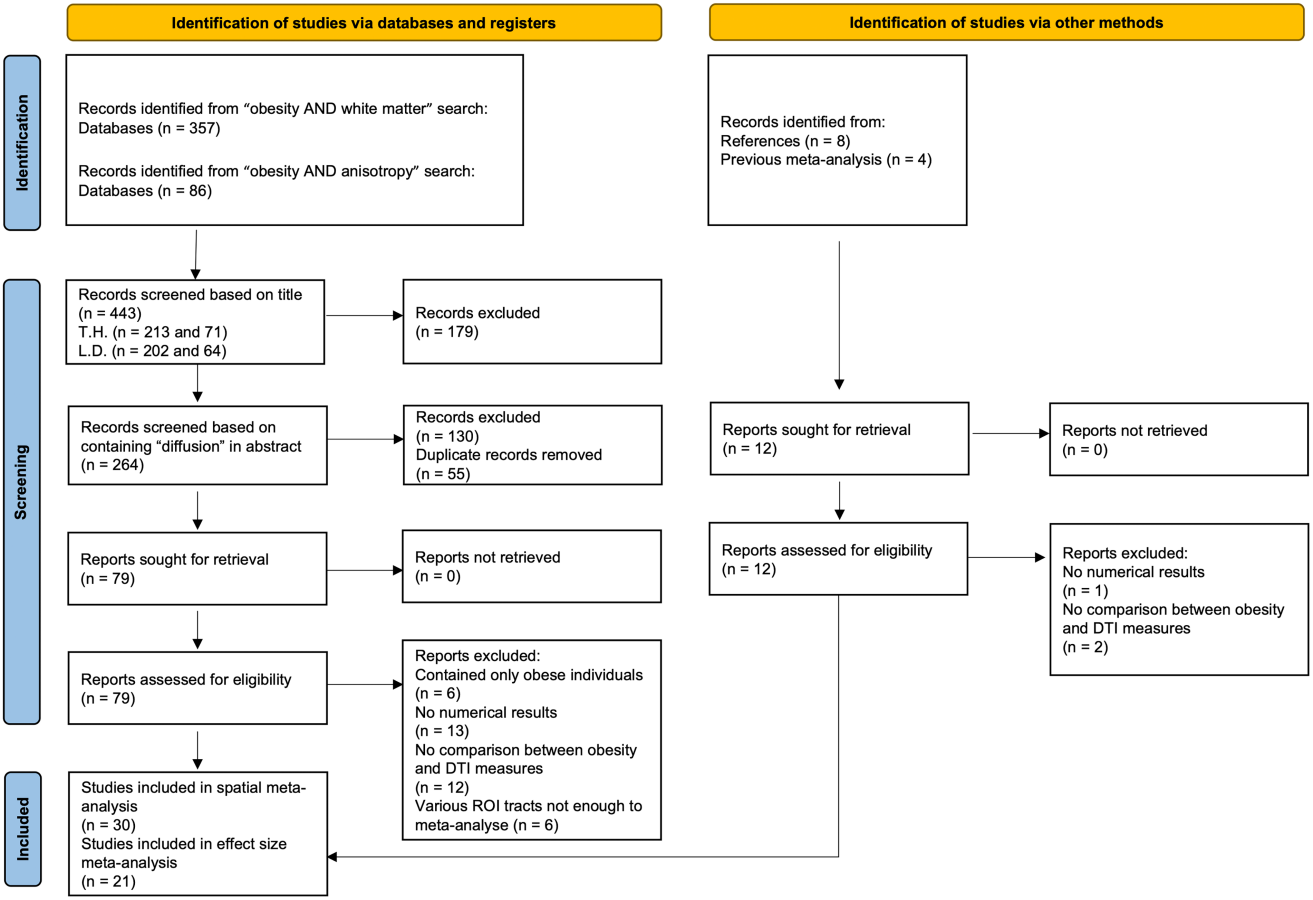
Study selection flow chart. Template adapted from Page et al. (27).

We followed the Preferred Reporting Items for Systematic Reviews and Meta-Analyses (PRISMA) statement (27) and performed a systematic search of articles until January 15, 2023 in PubMed database,^1^ using the following keywords: (1) “obesity AND white matter”; and (2) “obesity AND anisotropy.” We excluded animal studies and also searched references of downloaded articles and previous reviews (see Figure 1; Tables 1 and 2) and meta-analyses (26) for additional studies.

**Table 1:** Summary of the studies included in the spatial voxel-based meta-analysis.

	Name	Sample size	Number of obese	Number of non-obese	Measure used	Measure average/SD/range	Age (mean/range)	Co-morbid	TBSS (Y/N)	FA Obese (− > <)	MD Obese	AD Obese	RD Obese
Studies comparing overweight/obese versus normal weight individuals	Estella et al. (58)	30	13	17	BMI	33.64 ± 4.7; 22.50 ± 2.0	35.52/18 to 55	Binge eating disorder	Y	–	–	–	–
	Hidese et al. (40)	202	17	185	BMI	22.7 ± 4.3	40.65/16 to 64	Major depressive disorder	Y	<	N/A	N/A	N/A
	Karlsson et al. (59)	45	23	22	BMI	47.29 ± 3.74; 24.02 ± 2.28	46.88/N/A		N	<>	<>	N/A	N/A
	Kullmann et al. (60)	48	24	24	BMI	33.16 ± 3.16; 28.13 ± 1.38; 22.43 ± 1.61	26.56/21 to 37		N	<	<>	<>	<
	Lou et al. (61)	49	22	27	BMI	31.44 ± 3.34; 21.54 ± 2.06	30.38/N/A	Elevated Plasma LDL	Y	<	N/A	N/A	N/A
	Mazza et al. (62)	164	80	84	BMI	29.23 ± 3.91; 21.99 ± 2.10	46.97/N/A	Bipolar depression	Y	<	>	>	>
	Nouwen et al. (63)	33	13	20	BMI-z	3.11 ± 0.65; 0.32 ± 0.98	15.7/12 to 18	Type 2 diabetes	Y	-	-	-	-
	Ottino-González et al. (54)	52	21	31	BMI	30.75 (4.86) 25.20–49.69; 22.35 (2.01) 19.00–24.99	30.54/21 to 40	Allostatic load	Y	-	-	-	-
	Papageorgiou et al. (64)	172	52	120	BMI	N/A	9.45/8 to 10		Y	<	-	-	-
	Reyes et al. (43)	76	45	31	BMI	29.9 ± 3.3; 22.5 ± 1.6	22.35/N/A		Y	<	-	-	-
	Rice et al. (65)	30	15	15	N/A	N/A	21/17 to 30	Prader Willi Syndrome	Y	<	N/A	N/A	N/A
	Ryan and Walther (66)	94	42	52	BMI	34.9 ± 3.3; 27.6 ± 1.4; 22.3 ± 1.6	69.33/52 to 92		N	<	N/A	<	-
	Samara et al. (67) 1	46	25	21	BMI	40 ± 4.9; 22 ± 2.2	29.8/N/A		Y	<	-	<	-
	Samara et al. (67) 2	59	18	41	BMI	35.7 ± 4.3; 21.7 ± 1.7	29.65/N/A		Y	-	-	<	-
	Segura et al. (68)	38	19	19	Waist circumference	105.82 ± 8.78; 84.71 ± 8.54	60.45/50 to 80	Metabolic Syndrome	N	<	N/A	N/A	N/A
	Shott et al. (69)	42	18	24	BMI	34.78 ± 4.44; 21.64 ± 1.26	28.05/N/A		N	<	N/A	N/A	N/A
	Spangaro et al. (70)	88	54	34	BMI	29.55 ± 3.61; 22.30 ± 2.06	36.67/20 to 50	Schizophrenia	Y	-	-	<	-
	van Bloemendaal et al. (71)	30	15	15	BMI	32.6 ± 0.8; 23.4 ± 0.4	58.8/N/A	Type 2 diabetes	Y	–	–	–	–
	Zhang et al. (50)	33	15	18	BMI	38.10 ± 1.50; 21.60 ± 0.70	27/N/A	Morbid Obesity	Y	<	>	N/A	N/A
Studies investigating an association between an obesity-related measurement as a continuous variable and DTI measures	Alarcón et al. (72)	152	64	88	Age-Adjusted BMI Percentile	30.9 (5.4); 24.2 (1.6); 20.3 (1.9)	14.1/12 to 17		Y	<	-	<	>
	Cárdenas et al. (73)	23	13	10	VAT	427.65 (194.01), 238 to 869; BMI 25.48 (2.49), 22 to 33	37.22/25 to 52		Y	<	-	-	-
	Dennis et al. (74)	499	N/A	N/A	BMI	23.31, 16 to 38	23.8/20 to 30	NEGR1 genotypes	N	-	-	-	-
	Figley et al. (75)	32	N/A	N/A	BMI	24.85, 18 to 37	29.8/18 to 57		N	<>	-	N/A	N/A
	He et al. (76)	266	15	251	BMI	20.4 (2.2), 16.3 to 27.8	20.38/18 to 24		Y	<	N/A	N/A	N/A
	Koivukangas et al. (77)	40	8	32	BMI	N/A	22.25/20 to 25	Risk of psychosis	Y	<	-	-	>
	Repple et al. (78) MNC	369	N/A	N/A	BMI	24.65 ± 4.08, 18.17 to 42.21	39.39/18 to 59		Y	<	N/A	N/A	N/A
	Repple et al. (78) HCP	1,064	N/A	N/A	BMI	26.40 ± 5.1, 16.48 to 47.76	28.75/22 to 37		Y	<	N/A	N/A	N/A
	Verstynen et al. (79)	155	N/A	N/A	BMI and waist circumference	27.15 ± 4.82, 18.5 to 42.3	40.7/30 to 50		N	<	N/A	N/A	N/A
	Xu et al. (80)	51	29	22	BMI	30.8 (4.7); 23.0 (1.4)	29.6/N/A		Y	<	>		>
	Zhang et al. (28)	1,255	N/A	N/A	BMI	25.82 (3.69), 16.81–50.15	55.43/19 to 80		Y	<	N/A	N/A	N/A

Symbols –, >, and < indicate the direction of results, where – is no difference, < means obese is lower than healthy weight, > means obese is higher than healthy weight, and N/A means no results were reported.

**Table 2:** Summary of the studies included in the region of interest effect size meta-analysis.

	Name	Sample size	Number of obese	Number of non-obese	Measure used	Measure average/SD/range	Age (mean/range)	Co-morbid	TBSS (Y/N)	FA obese (− > <)	MD obese	AD obese	RD obese
Studies comparing overweight/obese versus normal weight individuals	Augustijn et al. (81)	43	18	25	BMI	31.03 (4.62), 16.90 (1.15)	9.5, 7 to 11		N	<	–	N/A	N/A
	Hidese et al. (40)	114	7	107	BMI	22.7 (4.3), 22.3 (3.5)	40.65, 16 to 64	Major despressive disorder	Y	<	N/A	N/A	N/A
	Lukoshe et al. (82)	74	13	61	N/A	N/A	12.63, 6 to 25	Prader-Willi syndrome	Y	<	>	–	<−
	Moreno-Navarrete et al. (41)	44	25	19	Body composition	43.1 (5.7), 23.9 (3.2)	49.25, 30 to 65		Y	<	N/A	<	<
	Reijmer et al. (42)	70	35	35	BMI	28 (3), 26 (3)	71.05, 65 to 80	Type 2 diabetes	N	<	>	N/A	N/A
	Stanek et al. (83)	72	17	55	BMI	34.14 (4.57), 22.71 (1.54)	45.14, 21 to 86	Depression	Y	<	N/A	N/A	N/A
	Steward et al. (84)	49	24	25	BMI	42.67 (7.11), 20.89 (1.87)	35, N/A		N	<	>	N/A	N/A
	Tang et al. (85)	106	74	32	BMI	29.41 (6.27), 27.57 (4.27)	46.98, N/A	Schizophrenia	N	–	N/A	N/A	N/A
	Yamada et al. (86)	16	8	8	N/A	N/A	19, 8 to 29	Prader-Willi syndrome	N	<	N/A	N/A	N/A
Studies investigating an association between an obesity-related measurement as a continuous variable and DTI measures	Alosco et al. (87)	120	35	85	BMI	18.92 (2.33), 27.14 (3.46)	13.54, 6 to 18		Y	–	N/A	N/A	N/A
	Bettcher et al. (88)	151	N/A	N/A	BMI	25.4 (3.7)/18.0 to 37.2	71.6, 62 to 87		Y	<	N/A	N/A	N/A
	Bolzenius et al. (39)	62	38	24	BMI	25.74 (3.72)/18.60 to 33.45	62.4, 51 to 81		N	<	N/A	N/A	N/A
	Byeon et al. (89)	264	176	88	BMI	22.50 (1.64), 35.10 (4.58)	38.19, N/A		N	<	>	N/A	N/A
	Carbine et al. (90)	87	40	47	BMI	55.23% (22.71%), 93.65% (4.29%)	16.42, 12 to 20		N	<>	no numerical	no numerical	no numerical
	Dekkers et al. (18)	12,087	7,358	4,703	BMI	26.6 (4.4)	62, 45 to 76		N	<	>	N/A	N/A
	Metzler-Baddeley et al. (91)	38	N/A	N/A	BMI	24.9 (3.5)/17.5 to 32.5	67.9, 53 to 93		N	–	>−	>−	>−
	Mueller et al. (92)	23	N/A	N/A	BMI	29.1 (7.0)	26.4, N/A		Y	<	N/A	<	>
	Pines et al. (93)	77	N/A	N/A	BMI	34.92 (4.08)	50.8, 22 to 76	Depression	N	–	N/A	N/A	N/A
	Rodrigue et al. (94)	1,320	N/A	N/A	BMI	31.6 (7.4)	42, 18 to 97	Inflammatory markers	Y	<	N/A	N/A	N/A
	Williams et al. (95)	665	150	515	BMI	N/A	71.3, 50 to 95	APOE ε4	N	<	>	N/A	N/A
	Zhang et al. (28)	1,255	N/A	N/A	BMI	25.82 (3.69)/16.8 to 50.2	55.43, 19 to 80		Y	<	N/A	N/A	N/A

Symbols –, >, and < indicate the direction of results, where – is no difference, < means obese is lower than healthy weight, > means obese is higher than healthy weight, and N/A means no results were reported.

### 2.2. Eligibility criteria

Only studies that included either whole brain analyses using voxel-based morphometry (VBM) or tract based spatial statistics (TBSS) were used for the voxel-based spatial meta-analysis, while studies providing numerical estimates of effect sizes of fractional anisotropy (FA) from region of interest (ROI) analyses were used for the ROI effect size meta-analysis. We originally planned to additionally look at mean diffusivity (MD), axial diffusivity (AD), and radial diffusivity (RD) but there were too few studies to analyze.

We included studies which analyzed associations between FA and a measure of obesity, including body mass index (BMI), waist-to-hip ratio, waist circumference, body composition (total fat mass or body fat percentage), and body fat distribution as measured by abdominal MRI scans. Some studies compared groups of people with overweight or obesity and normal weight individuals, while others investigated an association between an obesity-related measurement (e.g., BMI, waist circumference, waist-to-hip ratio, total body fat mass, percentage of body fat mass, visceral adipose tissue) as a continuous variable and diffusion tensor imaging (DTI) measures. We jointly analyzed results from both types of analyses.

Our objective was to maximize the scope of literature included in the analyses, and so we set minimum exclusion criteria. Exclusion criteria were set to studies that did not age-match participants, did not test for association between obesity and DTI measures, did not provide numeric results, or did not respond to email requests for data availability. All the whole brain studies provided corrected results.

The initial search identified 443 studies, and two raters, TH and LD independently screened the list of titles and abstracts for inclusion, see Figure 1 for an overview of the study selection.

### 2.3. Spatial voxel-based meta-analysis

We used the Anisotropic Effect Size Seed-based d Mapping (AES-SDM) method^2^ (22, 23) for the spatial voxel-based meta-analysis and extracted peak coordinates (*x*, *y*, *z*) and corresponding *t*-statistics from each study. If the study included a different measure of effect size like a value of *p* or *z*-score, these were converted to *t*-statistics using the SDM software. We contacted the authors of studies that met our inclusion criteria for additional information. With this strategy, we obtained one full t-map (28), and used it jointly with the peak coordinates and *t*-values from other studies. Inclusion of t-maps improves precision of the results (22). See Table 1 for additional information about each study.

The AES-SDM method uses the peak coordinates and effect size from individual studies, to recreate, for each study, a map of the effect sizes of the statistical associations, and then conduct a standard random-effects variance-weighted meta-analysis in each voxel. This version uses anisotropic kernels, which assign different values to surrounding voxels of a peak coordinate based on spatial correlations between them (23). We assessed potential publication bias *via* AES-SDM software and used jack-knife analysis to determine the robustness of the results after removing individual studies from the meta-analysis. We preprocessed all data files with the TBSS template based on the FA skeleton (29) included in AES-SDM as it allows combination of VBM and TBSS studies (30). We set all statistical parameters as recommended (anisotropy 1.0, FWHM 20, mask TBSS, voxels 2 mm) and performed a 500-permutation randomization. After calculating meta-analytic means, we applied a combined threshold (*p* < 0.001, peak threshold > 1) as suggested by Radua et al. (22) but more conservative and discarded clusters comprising fewer than 15 voxels. This method showed an adequate sensitivity and an excellent control of false positives (31). Unlike other methods of coordinate based meta-analyses, the AES-SDM allows for inclusion of studies which showed no statistically significant results and models the relative increases and decreases in the same map. We used FSLeyes software^3^ to visualize effect sizes by overlaying our results with brain and FA skeleton templates (32). See Table 1. for a description of the studies included in the meta-analysis.

### 2.4. Effect size ROI meta-analysis

To validate the spatial voxel-based meta-analysis results we conducted an ROI effect size meta-analysis investigating ROI from the spatial meta-analysis and regions most explored in the literature (see Supplementary Table S1 online for a full list of ROI). We manually recorded Cohen’s *d* (standardized mean difference) effect size measures from individual studies (33). When studies included a different measure of effect size, this was converted to Cohen’s *d* by standard formula (24). We also recorded other relevant information like confidence intervals and measures of significance, along with sample descriptive statistics, see Table 2.

We used Comprehensive Meta-Analysis Software (CMA) version 3.3.070^4^ to conduct the ROI meta-analysis. The CMA software can convert different individual study information including effect size, variability, and significance variables of both categorical and continuous studies into one meta-analysis. We performed analyses for regions of interest that had four or more viable studies. Only three studies investigated the anterior thalamic radiation, but we analyzed this region to test for replication of the spatial meta-analysis results. To assess whether results may be driven by a few studies reporting very large effect sizes, we repeated the analyses excluding studies with effect sizes > 1. We Assessed potential publication bias using R 4.0.3 and the metaphor package (v3.0-2; https://www.metafor-project.org/doku.php/metafor). See Table 2 for a description of the studies used in this meta-analysis.

## 3. Results

### 3.1. Study characteristics

We included 30 studies in the voxel-based meta-analysis, and 21 in the ROI meta-analysis, see Tables 1 and 2. The spatial meta-analysis included 5,237 participants 8–92 years old, while there were 16,505 participants in the effect size meta-analysis (age range 6–95 years). Most studies reported a negative correlation between FA and obesity, i.e., decreased FA in overweight or obese individuals, in both the voxel-based (23 out of 30 studies) and ROI datasets (17 out of 21 studies). Obesity was mainly measured using BMI, or adjusted BMI measures, but other weight measures were not excluded. See Supplementary Tables S2 and S3 online for detailed description of the included studies.

### 3.2. Spatial voxel-based meta-analysis results

**Figure 2:**
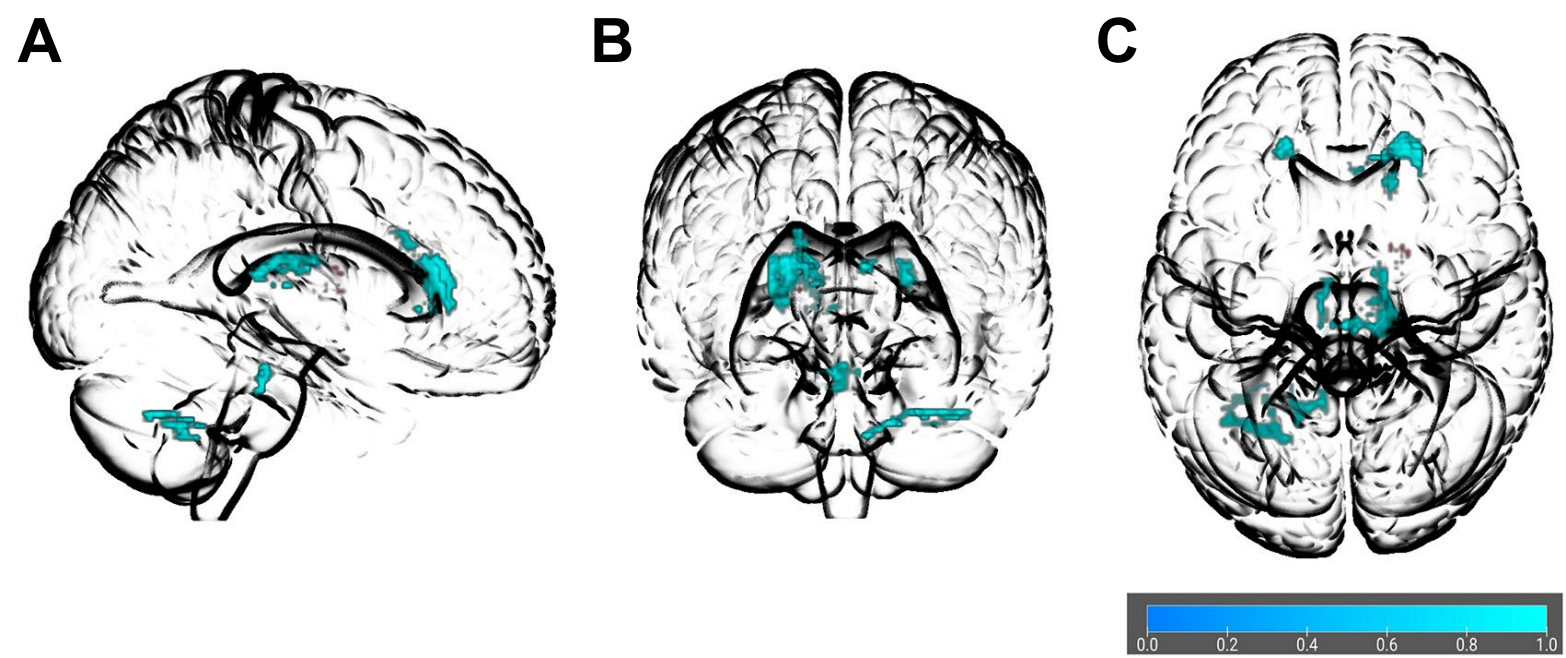
Glass brain of association between obesity and FA in the spatial voxel-based meta-analysis results. The orientations are **(A)** right sagittal, **(B)** anterior coronal, and **(C)** superior axial. Colored bar indicates values of 1–*p.*

The AES-SDM meta-analysis indicated that obesity measures were related to reduced FA values in several white matter regions, including the right and left genu of the corpus callosum (MNI = 22, 32, 12; MNI = −18, 30, 16), left splenium of the corpus callosum (MNI = −8, −28, 16), middle cerebellar peduncles (MNI = −38, −56, −38), anterior thalamic radiation (MNI = 14, −26, 12), right cortico-spinal projections (MNI = 4, −30, −24), and the left cerebellum (MNI = −16, −60, −40) see Table 3; Figure 2 for details. We did not find any region with increased FA in obesity. Jack-knife analysis reliably reproduced each cluster except the left cerebellum, which was present only in 8 out of 30 iterations (see Table 3). We did a subgroup analysis in adults where the corpus callosum (MNI = 22, 32, 12; *p* > 0.001), and middle cerebellar peduncles (MNI = −24, −66, −36; *p* > 0.001) clusters were replicated. No potential publication bias was detected except for the left cerebellum, where the Egger test was statistically significant (*p* = 0.042; see Table 3).

**Table 3:** Results of SDM spatial voxel-based meta-analysis.

Region	MNI coordinates							
	*X*	*Y*	*Z*	SDM-Z	*p*	Voxels	Jacknife	Egger test (Bias; *p*)
Genu corpus callosum	22	32	12	−2.503	0.00069	147	27/30	−0.62; 0.104
Middle cerebellar peduncles	−38	−56	−38	−2.711	0.00020	62	27/30	0.33; 0.336
Right anterior thalamic radiation	14	−26	12	−2.090	0.00191	70	27/30	0.01; 0.953
Right cortico-spinal projections	4	−30	−24	−2.741	0.00020	39	25/30	0.02; 0.943
Left cerebellum	−16	−60	−40	−2.591	0.00042	35	8/30	0.60; 0.042
Genu corpus callosum	−18	30	16	−1.983	0.00233	31	28/30	−0.36; 0.282
Splenium corpus callosum	−8	−28	16	−2.181	0.00177	28	27/30	0.11; 0.682

### 3.3. Region of interest meta-analysis results and validation of spatial meta-analysis findings

**Figure 3:**
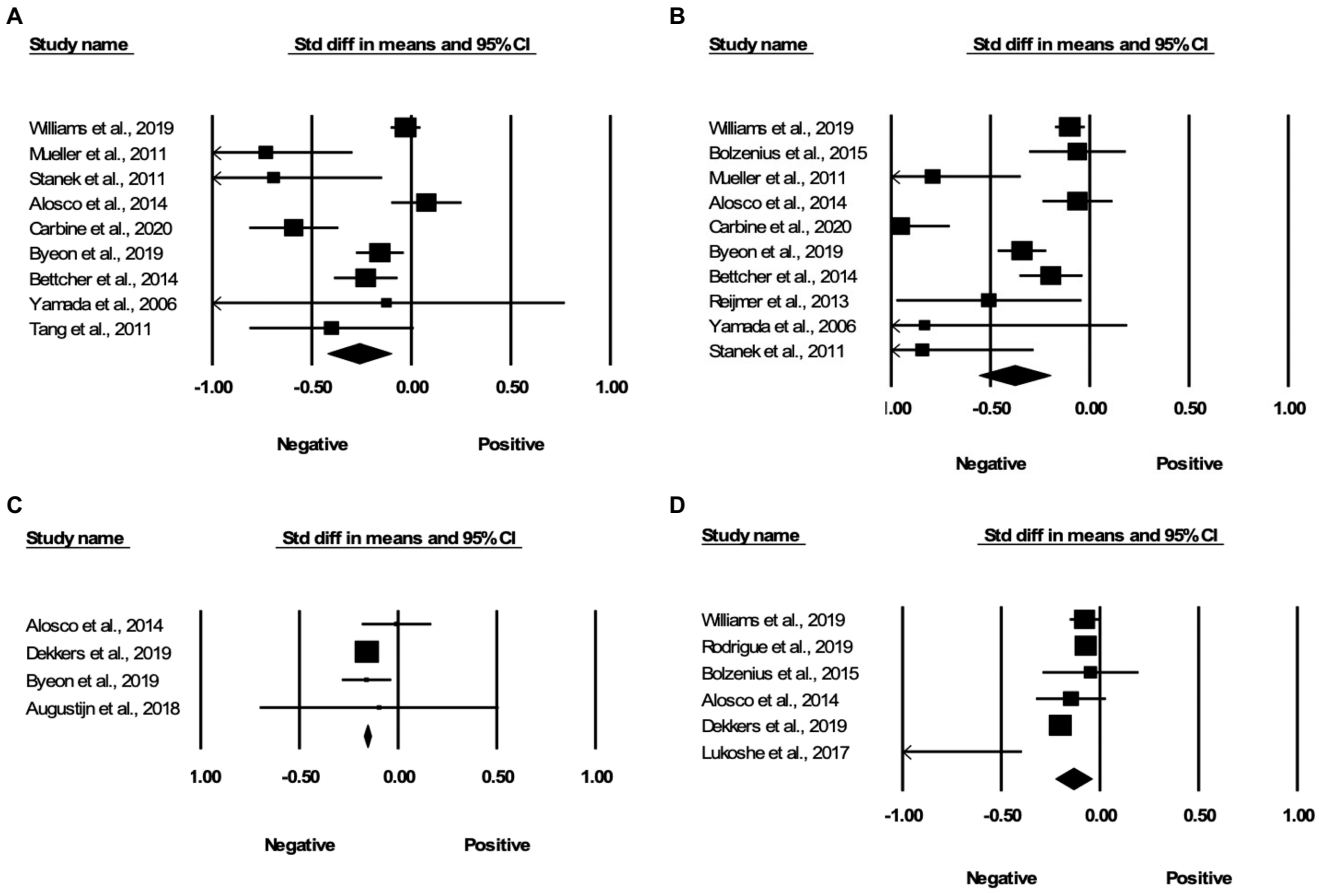
Associations between obesity and FA in the region of interest effect size meta-analyses. Forest plots of the significant findings from the effect size meta-analysis within the regions of **(A)** genu of the corpus callosum, **(B)** splenium of the corpus callosum, **(C)** middle cerebellar peduncles, and **(D)** superior longitudinal fasciculus.

Relative to controls, obese individuals had lower FA in some of the same regions as those identified in the spatial voxel-based meta-analysis, including the genu of the corpus callosum (Cohen’s *d* = −0.263, 95% Confidence Interval –0.423 to –0.103, *p* = 0.001; see Figure 3A), splenium of the corpus callosum (Cohen’s *d* = −0.380, 95% Confidence Interval –0.560 to –0.200, *p* < 0.001; see Figure 3B), and middle cerebellar peduncles (Cohen’s *d* = −0.157, 95% Confidence Interval-0.174 to-0.139, *p* < 0.001; see Figure 3C). We found two other ROIs, which showed significantly lower FA in obese individuals as compared to controls of a healthy weight and were not represented in the spatial voxel-based meta-analysis. These included the superior longitudinal fasciculus (Cohen’s *d* = −0.135, 95% Confidence Interval –0.227 to –0.044, *p* = 0.004; see Figure 3D), and the fornix which was not significant after removal of a study with a large effect size. Some regions which showed significant associations in spatial meta-analysis, including body of the corpus callosum, uncinate fasciculus, cingulum, parahippocampal cingulum, cortico-spinal tracts, anterior thalamic radiation, showed comparable FA values between people with overweight and obesity and controls in the ROI studies. We did not detect potential publication bias in any region except the splenium of the corpus callosum where the test for funnel plot asymmetry was significant (*z* = −2.175, *p* = 0.03).

## 4. Discussion

In this study, we conducted a spatial voxel-based meta-analysis on obesity and DTI from 30 individual studies to determine which white matter tracts were most frequently associated with obesity. We further validated the spatial voxel-based meta-analysis results in a ROI effect size meta-analysis of an additional 21 studies. The spatial meta-analysis suggested that overweight and obesity were associated with lower FA in the left and right genu of the corpus callosum, left splenium, middle cerebellar peduncles, anterior thalamic radiation, right cortico-spinal projections, and the left cerebellum. However, the left cerebellum changes were only reproduced in 8/30 iterations in the jack-knife analysis (see Table 3). The effect size meta-analysis results replicated the negative associations between overweight and obesity and FA in the genu and splenium of the corpus callosum, as well as the middle cerebellar peduncles. Additionally, we found that the superior longitudinal fasciculus, which was not represented in the spatial meta-analysis results, also showed reduced FA in people with overweight and obesity. The effect size of overweight and obesity related changes in these regions ranged from –0.135 to –0.38 (see Figure 3).

A recent spatial meta-analysis also investigated the association between obesity and white matter microstructure, however there were some differences between the present study and the previous meta-analysis. Our meta-analysis contained 30 studies, while the previous study contained 16 studies due to restrictive exclusion criteria limiting studies including children or individuals with psychiatric conditions (26). Obesity in the developed world typically starts early in childhood or adolescence (34, 35). Also, many patients who are overweight or obese will have co-morbid conditions such as anxiety, depression, diabetes, or metabolic syndrome (3, 4, 8–10), and excluding these individuals is not representative of the general population. In addition, the previous study performed a spatial voxel-based meta-analysis, whereas for the first time we performed a ROI effect size meta-analysis for validation of the spatial voxel-based meta-analysis results. The ROI effect size meta-analysis contained an additional 21 studies with a range of regions including a breakdown of the corpus callosum and other regions not shown in the spatial analysis, with a combined total of 16,505 participants. We replicated the results of the previous spatial meta-analysis, finding reduced FA values for the genu of the corpus callosum in people with overweight or obesity as compared to healthy weight individuals, and expanded these findings to the splenium of the corpus callosum, and middle cerebellar peduncles which were both validated in the ROI effect size meta-analysis.

If we overlay the white matter changes found in this study with the obesity related gray matter changes from the previous meta-analysis, they fall in three distinct networks (17). The corticospinal tract from our spatial voxel-based meta-analysis, passes through the middle cerebellar peduncles as a part of the corticopontocerebellar pathway, (36) which may be linked to reduced exercise or mobility in obesity (37, 38). The splenium of the corpus callosum is implicated in the default mode network (39), which is involved at rest in self-referential processes (40). We also found significant association between obesity and reduced FA in the genu of the corpus callosum, which has afferent projections to the prefrontal cortex (41, 42) and is a part of the executive control network (17). The default mode network and the executive control network may be implicated in the cognitive deficits in obesity (37, 38, 43–45).

We cannot infer causality from cross-sectional studies. Reduced FA in specific regions of the brain may either predispose an individual to obesity, or the changes could be a consequence of obesity. The spatial location of changes could help us infer the direction of association. If obesity damages the brain, we would expect to find diffuse alterations. The white matter correlates of obesity in this meta-analysis were not diffuse, but rather localized to only a few tracts and a few specific networks. This may indicate that white matter changes in specific circuits involving executive functioning or default mode network predispose to obesity.

Evidence of reduced FA generally suggests water diffusion is isotropic, or the fiber bundles are less organized (46), but there are many other theories including a decrease in myelination of axons in white matter (47, 48) or edema, although this is unclear and mean diffusivity is a better predictor (49, 50). The mechanisms through which obesity contributes to these changes are unknown, and maybe multifold instead of a singular mechanism, but some plausible candidates include systemic inflammation (51), the overactivation of microglia (52, 53), stress (54), microvascular changes, insulin resistance, hyperglycemia, plasticity related to lower mobility, or genetic predisposition to brain changes which increase the risk of obesity.

The present study was a starting point to establish the location of brain changes in overweight and obesity and associated effect sizes of these differences. Due to the cross-sectional nature of this study, we cannot determine causality between brain changes and obesity or the underlying mechanisms. However, to determine causality, future studies should either employ a longitudinal design or a mendelian randomization study. An alternative is studying obesity in animals due to the ability to control their environment throughout the lifespan.

As with any meta-analysis, we are limited by the information provided in published studies and cannot control the reporting of results including those that might be negative and not published. We were not able to analyze MD, AD, and RD associations with overweight and obesity as these measures were reported only in a minority of studies. Of the 79 studies originally read to determine suitability for inclusion in this study, many articles did not meet the criteria for inclusion by not reporting the respective statistics, including the peak coordinates, t-values, or sample sizes. It is very important for future studies to maintain a high standard for reporting results and follow reporting guidelines (55, 56). There is an argument after seeing much variability in the pre-processing and processing of DTI data around the world for a standardizing pipeline as in the Enhancing NeuroImaging Genetics through Meta-Analysis (ENIGMA) consortium studies (57), which would remove some methodological variability. The methods of scanning had heterogeneity due to different magnet strengths, although many were 3.0 T, and different MRI manufacturers. Our meta-analyses contained varying ages from children to the elderly. Although FA is not reliable when comparing across wide age spans, this was less of a problem for this study as the studies were age matched, containing children with overweight or obesity comparing FA with children of a healthy weight, and other studies with older adults comparing similar age ranges. Most studies in the present meta-analyses primarily used BMI as a measure of obesity, however, a few studies used body composition, waist circumference, or visceral adipose tissue. It would be preferable to compare measures of obesity using the same metric, but we are limited to the methodology provided in previous studies. The clusters we found were small, but the highly localized nature of the findings could have interesting implications. We provided some plausible explanations for the results of these meta-analyses, but the underlying mechanisms of obesity, and inferring causality for specific FA differences are questions that cannot be answered *via* a cross-sectional design as in this study, as this would require a prospective study to look at changes over time.

A strength of this study is our replication and extension of the previous meta-analysis (26) results indicating association between overweight and obesity and lower FA in the genu of the corpus callosum in a larger sampling of studies, which was also extended to the middle cerebellar peduncles and splenium of the corpus callosum in our voxel-based meta-analysis and validated in the ROI effect size meta-analysis. Our combination of spatial voxel-based and ROI effect size meta-analysis allowed us to obtain information about location and extent of any changes, as well as to check for replication among different sets of studies. The replication of exploratory findings in the spatial voxel-based meta-analysis by ROI effect size meta-analysis suggested that some of our findings were robust and not false positive.

To conclude, we found replicated associations of lower FA in the genu and splenium of the corpus callosum as well as the middle cerebellar peduncles with overweight and obesity. The extent of these obesity related alterations was a small to medium effect, but the main findings were highly replicated across different studies and meta-analyses. Since we currently do not know the mechanism behind brain changes in overweight and obesity, future studies should determine whether lower FA in these regions are a consequence or cause of obesity. Ultimately, we would need prospective longitudinal designs to clarify this question.

## Data availability statement

The original contributions presented in the study are included in the article/Supplementary material, further inquiries can be directed to the corresponding author.

## Author contributions

LD and TH were responsible for designing the search strategy for relevant literature, identifying relevant articles, and screening articles based on title and abstract. LD was responsible for assessing articles for eligibility, extracting, and analyzing the data, interpreting results, creating the figures and tables, and writing the manuscript. SM was responsible for assisting in the data analysis. JR was responsible for assisting in the data analysis and interpretation. TH and JR contributed to the manuscript and provided feedback. All authors contributed to the article and approved the submitted version.

## Funding

This study was supported by funding from the Canadian Institutes of Health Research (142255 and 180449).

## Conflict of interest

The authors declare that the research was conducted in the absence of any commercial or financial relationships that could be construed as a potential conflict of interest.

## Publisher’s note

All claims expressed in this article are solely those of the authors and do not necessarily represent those of their affiliated organizations, or those of the publisher, the editors and the reviewers. Any product that may be evaluated in this article, or claim that may be made by its manufacturer, is not guaranteed or endorsed by the publisher.

## Footnotes

^1^https://pubmed.ncbi.nlm.nih.gov

^2^www.sdmproject.com

^3^https://git.fmrib.ox.ac.uk/fsl/fsleyes/fsleyes

^4^www.meta-analysis.com
